# Consumption of artificial sweeteners during pregnancy and the risk of overweight in the offspring

**DOI:** 10.1017/S0007114525000455

**Published:** 2025-04-14

**Authors:** Eva M. Gjørup, Bodil H. Bech, Sofie Stampe, Thorhallur I. Halldorsson, Anne A. Bjerregaard, Sjurdur F. Olsen, Per G. Ovesen, Magnus Leth-Møller

**Affiliations:** 1 Department of Clinical Medicine, Aarhus University, Aarhus, Denmark; 2 Department of Gynecology and Obstetrics, Aarhus University Hospital, Aarhus, Denmark; 3 Steno Diabetes Center Aarhus, Aarhus University Hospital, Aarhus, Denmark; 4 Department of Public Health, Aarhus University, Aarhus, Denmark; 5 Department of Epidemiology Research, Statens Serum Institut, Copenhagen, Denmark; 6 Faculty of Food Science and Nutrition, University of Iceland, Reykjavik, Iceland; 7 Centre for Clinical Research and Prevention, Copenhagen University Hospital – Bispebjerg and Frederiksberg Hospital, Copenhagen, Denmark; 8 Department of Nutrition, Harvard T.H. Chan School of Public Health, Boston, MA, USA; 9 Department of Public Health, University of Copenhagen, Copenhagen, Denmark

**Keywords:** Childhood obesity, Epidemiology, Longitudinal cohort study, Artificial sweeteners, Pregnancy, Maternal-fetal health, Fetal exposure

## Abstract

Artificial sweeteners are used to reduce energy intake, but studies suggest that consumption during pregnancy may impact the offspring’s risk of overweight. In this longitudinal cohort study, we aimed to examine the association between consumption of artificially sweetened or sugar-sweetened beverages during pregnancy and offspring overweight from birth to 18 years in the Danish National Birth Cohort. A total of 101 042 pregnancies were enrolled in the Danish National Birth Cohort from 1996 to 2002. Follow-up was conducted throughout pregnancy, childhood and adolescence. Additionally, 72 821 women completed an FFQ during pregnancy, reporting intake of beverages sweetened with artificial sweeteners or sugar. Offspring height and weight were obtained during childhood and adolescence. Multivariate logistic regression was performed to estimate the OR for overweight concerning maternal beverage consumption. Analyses were adjusted for risk factors for childhood overweight, including maternal age, pre-pregnancy BMI, physical activity and smoking in pregnancy, healthy eating index, paternal BMI, socio-economic status and duration of breastfeeding. We found increased odds of overweight in 7-, 11-, 14- and 18-year-old offspring whose mothers reported drinking ≥ 1 artificially sweetened beverage daily during pregnancy compared with no consumption (18 years: adjusted OR 1·26 (95 % CI 1·12, 1·42)). We found decreased adjusted odds of overweight in 11- and 18-year-old offspring whose mothers reported drinking ≥ 1 sugar-sweetened beverage daily during pregnancy compared with no consumption. We found that consumption of artificially sweetened beverages during pregnancy was associated with an increased risk of overweight in childhood and adolescence after adjustment for risk factors for childhood overweight.

Abbreviations: ASB, artificially sweetened beverages; DNBC, Danish National Birth Cohort; GW, gestational week; LGA, large for gestational age; SES, socio-economic status; SSB, sugar-sweetened beverages.

The rate of obesity has increased dramatically throughout the last decades^(1)^, posing a threat to public health due to the increased burden of associated comorbidities^(2,3)^. To avoid excessive weight gain, many people try to lower energy intake, for example, by substituting caloric sweeteners with artificial sweeteners. True artificial sweeteners are synthetic sweetening agents used in a wide range of ‘sugar-free’ (diet) food products and beverages^(4–6)^. However, recent research suggests that artificial sweetener intake is associated with obesity^(7,8)^, and the WHO now advises against the use of non-nutritive sweeteners (including both artificial sweeteners and natural sweeteners such as steviol glycosides) for weight loss purposes^(9)^. Some studies have shown impairment in insulin tolerance in aspartame-exposed rats independently of body fat composition^(10)^ and increased glucose intolerance in both mice and humans exposed to artificial sweeteners, partly mediated by changes in the gut microbiome^(11,12)^.

Childhood obesity is associated with an increased risk of metabolic and cardiovascular disease in adulthood^(13,14)^. Several parental and early-life factors have been linked to childhood obesity risk, including duration of breastfeeding^(15)^, smoking in pregnancy^(16)^, parental socio-economic status (SES)^(17)^, maternal pre-pregnancy BMI^(18)^ and gestational weight gain^(18)^. Obesity rates among children and adolescents are high globally^(19)^, but intervention strategies to limit weight gain in youth differ in effectiveness^(20)^, underscoring the need for primary prevention strategies.

Although the WHO does not recommend artificial sweetener consumption for weight management, pregnant women with obesity or diabetes are recommended to substitute sugar-sweetened beverages (SSB) with artificially sweetened beverages (ASB) to reduce weight gain during pregnancy in some countries^(21)^. However, ASB consumption in pregnancy has previously been associated with overweight in infancy, early childhood and mid-childhood^(22–24)^. Yet, there is conflicting evidence in the field^(25)^, as others have found no association^(26)^.

Several hypotheses have been proposed to explain the association between ASB consumption and overweight in childhood, including metabolic programming, influence on sweet taste preference and influence on the human microbiome and microbiome-dependent glycaemic response^(11,27,28)^. It has been demonstrated that artificial sweeteners can cross the placenta into the fetal circulation, highlighting the possibility of prenatal exposure^(29,30)^. However, the underlying mechanism for the association has not yet been established^(31)^. Moreover, postnatal exposure to artificial sweeteners through breast milk has been established^(32,33)^.

To expand the knowledge on the safety of ASB consumption during pregnancy regarding offspring obesity, we aimed to examine the association between ASB and SSB consumption during pregnancy and offspring overweight from birth to 18 years of age. Additionally, we aimed to evaluate this association with SSB consumption and compare the two groups.

## Methods

### Study design and data collection

In this nationwide cohort study, we utilised data from the Danish National Birth Cohort (DNBC),^(34)^ which prospectively enrolled 101 042 pregnant women from 1996 to 2002 to investigate health across generations. The women were followed throughout pregnancy and after birth. Data on the offspring were collected from birth until 18 years of age. The last data were collected in 2022.

The pregnant women were enrolled at their first antenatal visit with their general practitioner^(34)^. Pregnant women were asked to participate if they spoke Danish and if they wished to carry their child to term, regardless of ethnic and cultural background. The women who agreed to participate were asked to participate in four interviewer-assisted phone interviews: two during pregnancy scheduled in gestational week (GW) 12 and GW 30 and two after pregnancy when the offspring was approximately 6 and 18 months old. In GW 25, the women completed an FFQ^(35)^. The FFQ has been validated for different variables, but not in totality^(36–38)^. Information on birth outcomes was extracted from the Danish Medical Birth Register. Written questionnaires were distributed to the families at ages 7 years (2005–2010) and 11 years (2010–2014) and to the offspring at ages 14 years (2013–2017) and 18 years (2016–2022). The timeline for the data collection is visualised in online Supplementary eFig. S1.

### Population

**Fig. 1.**
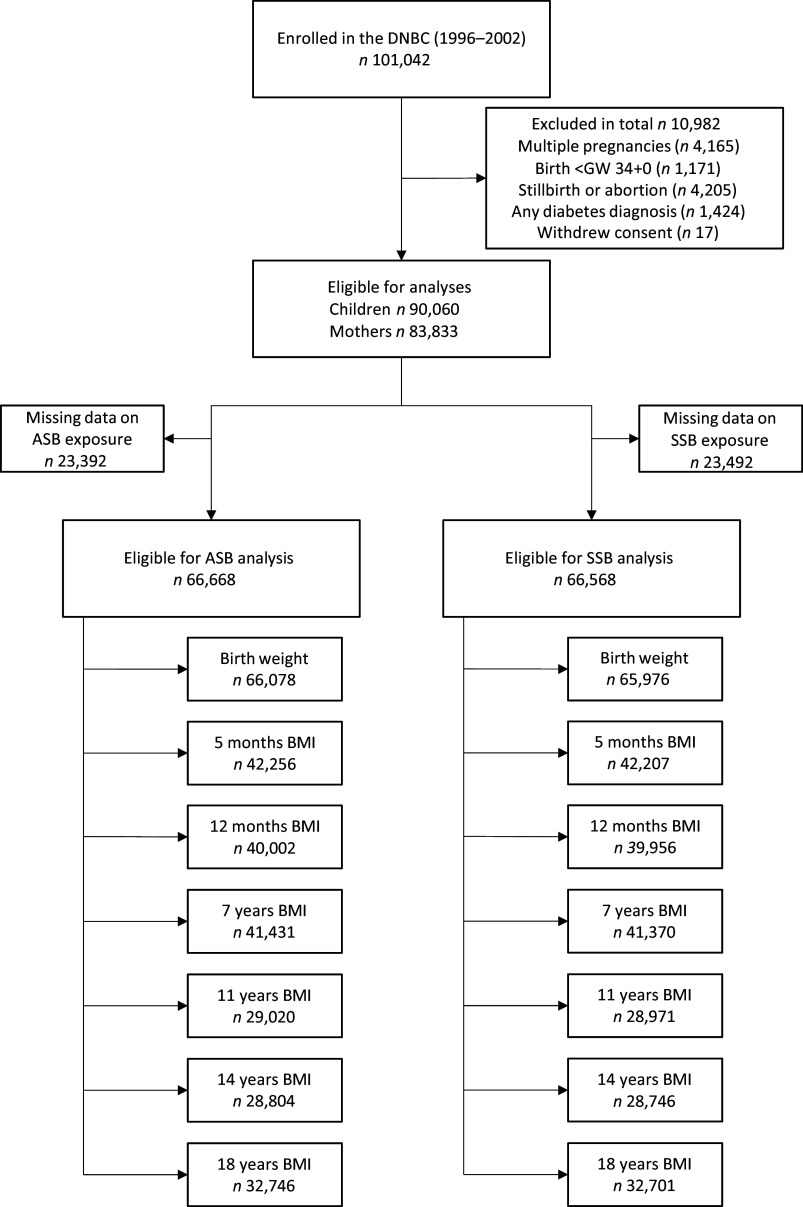
Flow chart for the inclusion process. DNBC, Danish National Birth Cohort; GW, gestational week; ASB, artificially sweetened beverages; SSB, sugar-sweetened beverages.

We included all singleton pregnancies that resulted in a live birth in GW 34 + 0 or later (Fig. 1). We included women who completed the FFQ with information on the consumption of ASB or SSB. We excluded women with diabetes, including those with pre-existing diabetes, newly detected manifest diabetes and gestational diabetes. Information on the diagnosis of diabetes was obtained from the Danish National Patient Register and based on ICD-8 (1977–1993) and ICD-10 codes (1994 onward). We excluded offspring with missing data on weight and length/height at each age of follow-up, but the offspring could be included in analyses for subsequent ages.

### Variables and data sources

#### Exposure

The exposures were defined as maternal consumption of ASB and SSB during pregnancy. In the FFQ completed in GW 25, the pregnant women were asked: ‘How many glasses/cups of the following beverages have you drunk within the last month?’ One glass/cup was defined as 250 ml. The mothers were asked to indicate the number of glasses consumed of sugar-sweetened and artificially sweetened (diet) carbonated and non-carbonated beverages. They could choose a number per month (none, 1, 2–3), per week (1–2, 3–4, 5–6) or per d (1, 2–3, 4–5, 6–7, 8 or more).

The consumption of ASB was calculated by adding the frequency of artificially sweetened carbonated and non-carbonated beverages. Similar calculations were made for SSB. Frequency of consumption of ASB and SSB was grouped into four exposure groups: never consumption, less than 1 beverage (250 ml) per week, 1–6 beverages per week and 1 or more beverages per d.

#### Outcome

The primary outcome was large for gestational age (LGA) birthweight and overweight at 5 and 12 months and 7, 11, 14 and 18 years. Length and weight measurements of the offspring in infancy (5 and 12 months) were done by the general practitioner at the 5- and 12-month health visits. The mothers reported these measurements during the last phone interview when the offspring was 18 months old. The measurements throughout childhood and adolescence were done by the parents or the offspring themselves and provided in the written questionnaires at ages 7, 11, 14 and 18 years. To avoid outliers, we excluded values of more than 5 sd from the mean, in either direction (*n* 489).

The offspring were defined as LGA if their birth weight was above 2 sd with the study population as internal reference material. Postnatal outcomes were based on offspring BMI reported at ages 5 and 12 months and 7, 11, 14 and 18 years. The cut-off value for overweight was BMI + 1·30 sd for boys and BMI + 1·19 sd for girls^(39)^. BMI z-scores were calculated by estimating mean (with sd) BMI dependent on age and sex using linear regression. We then estimated z-scores by subtracting the mean predicted BMI from the individual measured BMI and dividing it by the sd.

#### Covariates

Several covariates, all potential determinants of childhood obesity, were included in the analyses to control potential confounding and minimise bias (online Supplementary eFig. S2). Maternal age was retrieved from the Danish Civil Registration System^(40)^. Maternal pre-pregnancy BMI and physical activity in early pregnancy were provided in the first phone interview (GW 12). The women were asked whether they did any physical activity during pregnancy (yes/no). Gestational weight gain (kg) was provided by the mothers in the third interview. If the women confirmed smoking during pregnancy in any of the first three phone interviews, they were counted as having smoked during pregnancy (yes/no).

The maternal healthy eating index (points/80) was calculated from the FFQ^(41)^. The diet was rated using a score of 0–80, with 80 being the healthiest. It should be noted that SSB is included in the healthy eating index. The amount of each food item consumed was calculated by multiplying the frequency of consumption, as reported in the FFQ, with standard portion sizes. Total energy intake was then estimated by multiplying the amount consumed with the energy content of food as reported in the food composition tables^(35)^. Paternal BMI was reported in the 4th interview. Duration of breastfeeding was reported in the two postpartum phone interviews. Two variables were created: one describing breastfeeding for more or less than 4 months used for the analysis at 5 months and one describing breastfeeding for more or less than 6 months used for the 12-month and subsequent analyses. The mother’s occupational status defined her SES. If unavailable, the father’s occupational status was used instead. Socio-economic status was grouped into categories: unskilled, other work or receives public benefits; working class, craftsmen, short education or under education; and leaders or long/medium-long education. The criteria for the categorisation are described in further detail elsewhere^(42)^.

### Statistical analysis

For baseline characteristics, the mean (sd) was calculated for continuous variables and frequencies (%) for categorical variables. The associations between ASB or SSB consumption in pregnancy and offspring overweight were assessed using multiple logistic regression using ‘no consumption’ as a reference. To limit potential confounding, we adjusted for pre-pregnancy BMI, maternal age, maternal healthy eating index, physical activity in early pregnancy, smoking in pregnancy, combined SES, paternal BMI and breastfeeding duration. To evaluate associations between ASB consumption during pregnancy and birthweight and BMI z-scores as continuous measures, we used multiple linear regression to estimate the mean difference and 95 % CI. All analyses were performed as complete case analyses.

A few sub-analyses were performed: one analysis exchanged the maternal healthy eating index with total energy intake, and another analysis included additional adjustment for gestational weight gain. In addition, we designed an isoenergetic substitution model including the same covariates as the main analysis, with total energy intake replacing healthy eating index and with mutual adjustment of the exposures, where ASB was adjusted for SSB and SSB was adjusted for ASB. All analyses were performed as complete case analyses.

All data analysis was performed using R for Windows (version 4.3.1 (2023) (R Foundation for Statistical Computing).

## Results

A total of 101 042 pregnancies were enrolled in the DNBC. Of these, 90 060 children born to 83 833 women were eligible to participate in the study; 66 668 women had information on ASB from the FFQ, and 66 568 women had information on SSB from the FFQ (Fig. 1). Pregnant women consuming ≥ 1 ASB/d were younger, had higher BMI, more often smoked and had a lower SES compared with pregnant women with no ASB consumption (Table 1). There was no difference in healthy eating index, offspring sex, birth weight or gestational age at birth. Physical activity in early pregnancy was highest in the < 1 ASB/week group. Women with high ASB intake tended to have a shorter breastfeeding duration compared with the never group.

**Table 1.** Characteristics for all live born, singleton offspring born after gestational week 34 to mothers without diabetes in the Danish National Birth Cohort (Mean values and standard deviations; numbers and percentages)

	Total			Maternal ASB intake									Maternal SSB intake								
Characteristic	*n*	*n* 90 293*		*n*	Never, *n* 34 910*		< 1/week, *n* 6990*		1–6/week, *n* 13 636*		≥ 1/d, *n* 11 313*		*n*	Never, *n* 5875*		< 1/week, *n* 8285*		1–6/ week, *n* 29 143*		≥ 1/d, *n* 23 443*	
		Mean	sd		Mean	sd	Mean	sd	Mean	sd	Mean	sd		Mean	sd	Mean	sd	Mean	sd	Mean	sd
Maternal age	90 293	30·4	4·3	66 849	30·8	4·3	30·2	4·2	29·9	4·1	29·7	4·1	66 746	30·8	4·4	30·8	4·4	30·4	4·2	30·1	4·2
Pre-pregnancy BMI	83 195	23·5	4·3	63 079	22·9	3·8	23·4	4·0	24·0	4·3	24·6	5·0	62 975	24·2	5·0	23·5	4·2	23·5	4·2	23·3	4·0
Healthy eating index, points/80	68 955	23	7	66 692	23	7	23	7	23	7	22	7	66 647	28	7	26	7	23	6	20	6
Total energy intake, kcal/d	66 590	2471	683	64 334	2510	675	2390	669	2406	637	2507	713	64 249	2246	645	2278	603	2407	610	2689	696
		*n*	%		*n*	%	*n*	%	*n*	%	*n*	%		*n*	%	*n*	%	*n*	%	*n*	%
Smoking in pregnancy	88 457	23 306	26 %	66 612	8418	24 %	1497	22 %	3273	24 %	3095	27 %	66 502	1429	24 %	1706	21 %	6673	23 %	6393	27 %
Socio-economic status	84 249			63 839									63 736								
Unskilled, other work or receives public benefits		3331	4·0 %		1060	3·2 %	216	3·2 %	456	3·5 %	491	4·5 %		218	3·9 %	281	3·6 %	853	3·1 %	841	3·8 %
Working class, craftsmen, short education or under education		24 449	29 %		8371	25 %	1724	26 %	3899	30 %	3855	36 %		1683	30 %	2118	27 %	7409	27 %	6559	29 %
Leaders or long/medium-long education		56 469	67 %		23 833	72 %	4740	71 %	8686	67 %	6508	60 %		3700	66 %	5512	70 %	19 585	70 %	14 977	67 %
Maternal SSB intake	67 032			65 712																	
< 500 ml/week		23 833	36 %		9250	27 %	3009	44 %	5633	42 %	5559	51 %									
500–1500 ml/week		18 335	27 %		9826	28 %	1832	27 %	4183	31 %	2210	20 %									
≥ 1500 ml/week		24 864	37 %		15 450	45 %	2036	30 %	3510	26 %	3214	29 %									
Maternal ASB intake	67 005												65 590								
< 500 ml/week		47 644	71 %											2537	44 %	5549	68 %	20 812	72 %	17 916	78 %
500–1500 ml/week		7585	11 %											930	16 %	983	12 %	3747	13 %	1731	7·6 %
≥ 1500 ml/week		11 776	18 %											2345	40 %	1670	20 %	4169	15 %	3201	14 %
Paternal BMI	62 047	25·2	3·2	48 661	24·97	3·18	25·04	2·98	25·27	3·12	25·53	3·38	48 601	25·30	3·34	25·08	3·37	25·11	3·11	25·13	3·17
		*n*	%		*n*	%	*n*	%	*n*	%	*n*	%		*n*	%	*n*	%	*n*	%	*n*	%
Sex, boy	90 293	46 232	51 %	66 849	17 784	51 %	3589	51 %	7111	52 %	5753	51 %	66 746	2964	50 %	4243	51 %	14 985	51 %	11 987	51 %
		Mean	sd		Mean	sd	Mean	sd	Mean	sd	Mean	sd		Mean	sd	Mean	sd	Mean	sd	Mean	sd
Birth weight, g	89 897	3602	537	66 566	3604	532	3609	529	3613	539	3605	538	66 466	3599	533	3599	523	3610	529	3608	542
Gestational age, weeks	90 288	40·06	1·48	66 848	40·07	1·47	40·09	1·48	40·07	1·48	40·04	1·50	66 745	40·05	1·50	40·07	1·48	40·08	1·46	40·06	1·49
		*n*	%		*n*	%	*n*	%	*n*	%	*n*	%		*n*	%	*n*	%	*n*	%	*n*	%
Physical activity in early pregnancy	84 537	31 133	37 %	64 033	12 513	37 %	2793	42 %	5190	40 %	4081	37 %	63 930	2377	42 %	3318	42 %	10 937	39 %	7886	35 %
Breastfeeding duration	65 132			50 888									50 820								
≤ 6 months		26 433	41 %		9571	36 %	2007	38 %	4486	43 %	4162	49 %		1950	44 %	2431	38 %	8634	39 %	7173	40 %
> 6 months		38 699	59 %		17 024	64 %	3340	62 %	5937	57 %	4361	51 %		2445	56 %	3937	62 %	13 652	61 %	10 598	60 %
		Mean	sd		Mean	sd	Mean	sd	Mean	sd	Mean	sd		Mean	sd	Mean	sd	Mean	sd	Mean	sd
Timing of introduction of solid foods, months	66 126	4·44	0·70	51 211	4·48	0·70	4·45	0·68	4·41	0·69	4·34	0·67	51 164	4·42	0·71	4·47	0·71	4·45	0·69	4·42	0·69

ASB, artificially sweetened beverages; SSB, sugar-sweetened beverages.

* Mean (sd); *n* (%).

**Fig. 2.**
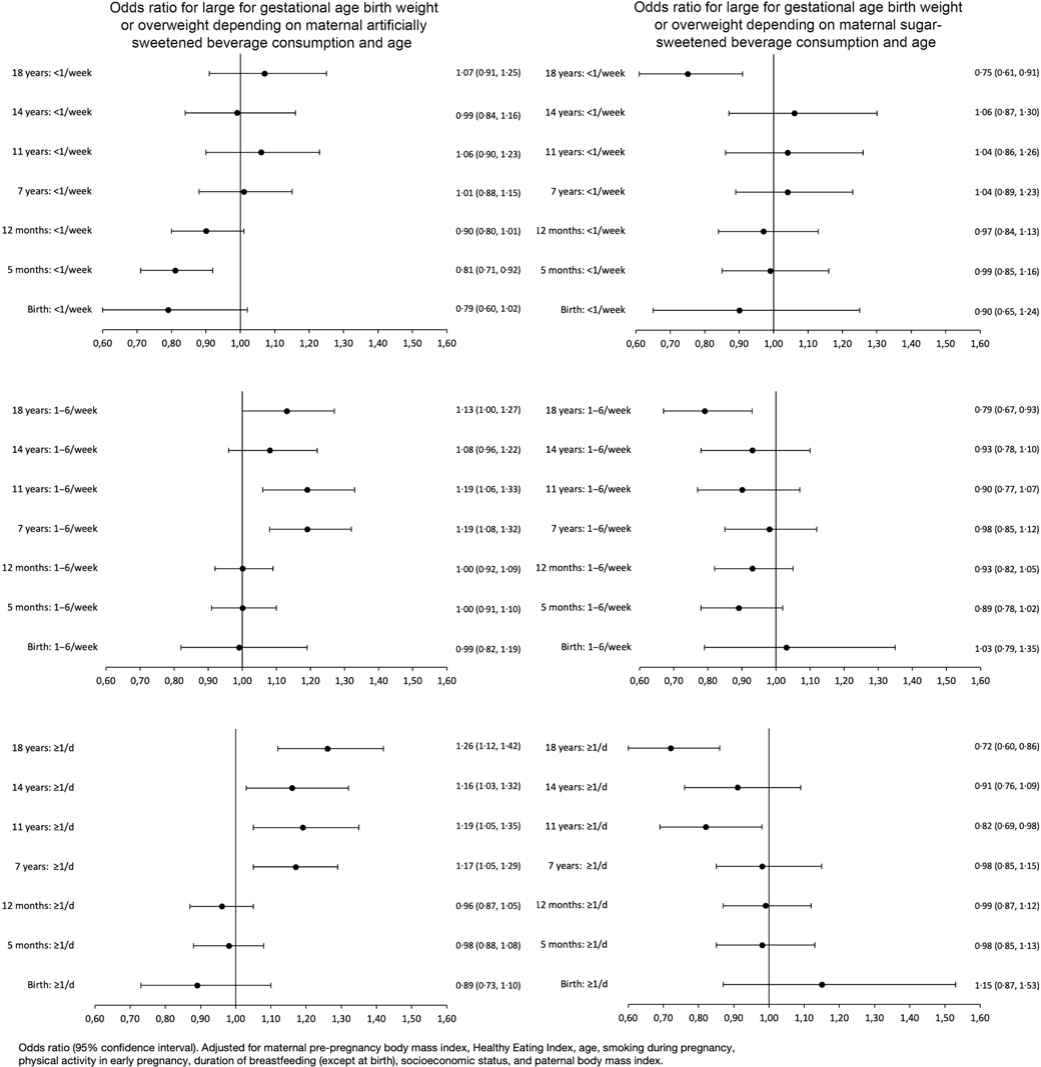
Forest plots summarising adjusted odds ratios for large for gestational weight birth weight or overweight depending on maternal artificially sweetened beverage consumption and age. The adjustment model included maternal pre-pregnancy BMI, healthy eating index, age, smoking during pregnancy, physical activity in early pregnancy, duration of breastfeeding (except at birth), socio-economic status and paternal BMI.

The associations between maternal ASB or SSB consumption and childhood overweight and LGA at birth are shown in Table 2 and Fig. 2. At birth and in infancy (5 and 12 months), we found no association between daily ASB consumption and LGA or overweight after adjustment. At 7, 11 and 18 years, we found higher odds of overweight in offspring whose mothers consumed 1–6 ASB/week or ≥ 1 ASB/d compared with no consumption. The associations attenuated but remained after adjustment (18 years in the ≥ 1 ASB/d group: adjusted OR 1·26 (95 % CI 1·12, 1·42)). At 14 years, we found higher odds for overweight in the ≥ 1 ASB/d group compared with never consumption, but not in the 1–6 ASB/week group. Daily maternal SSB consumption in pregnancy entailed lower odds for overweight at 11 years and 18 years compared with no consumption in both crude and adjusted analyses.

**Table 2.** Gestational age birth weight or overweight at different ages dependent on maternal beverage consumption during pregnancy (Odds ratios and 95 % confidence intervals)

		ASB consumption						SSB consumption					
Age	Consumption frequency	*n*	Unadjusted		*n*	Adjusted^†^		*n*	Unadjusted		*n*	Adjusted^†^	
			OR	95 % CI*		OR	95 % CI*		OR	95 % CI*		OR	95 % CI*
LGA at birth		66 078			45 690			65 976			45 656		
	Never		–			–			–			–	
	< 1/week		0·92	0·73, 1·13		0·79	0·60, 1·02		0·86	0·65, 1·13		0·90	0·65, 1·24
	1–6/week		1·12	0·96, 1·31		0·99	0·82, 1·19		0·94	0·76, 1·18		1·03	0·79, 1·35
	≥ 1/d		1·08	0·91, 1·28		0·89	0·73, 1·10		1·01	0·81, 1·28		1·15	0·87, 1·53
5 months		42 256			32 049			42 207			32 020		
	Never		–			–			–			–	
	< 1/week		0·85	0·77, 0·95		0·81	0·71, 0·92		0·96	0·84, 1·09		0·99	0·85, 1·16
	1–6/week		1·05	0·97, 1·14		1·00	0·91, 1·10		0·86	0·77, 0·97		0·89	0·78, 1·02
	≥ 1/d		1·05	0·97, 1·15		0·98	0·88, 1·08		0·92	0·83, 1·04		0·98	0·85, 1·13
12 months		39 361			35 988			39 320			35 969		
	Never		–			–			–			–	
	< 1/week		0·94	0·84, 1·05		0·90	0·80, 1·01		0·94	0·82, 1·07		0·97	0·84, 1·13
	1–6/week		1·08	1·00, 1·18		1·00	0·92, 1·09		0·89	0·79, 1·00		0·93	0·82, 1·05
	≥ 1/d		1·07	0·98, 1·17		0·96	0·87, 1·05		0·90	0·81, 1·02		0·99	0·87, 1·12
7 years		41 431			30 646			41 370			30 611		
	Never		–			–			–			–	
	< 1/week		1·10	0·98, 1·22		1·01	0·88, 1·15		0·84	0·73, 0·96		1·04	0·89, 1·23
	1–6/week		1·34	1·23, 1·45		1·19	1·08, 1·32		0·79	0·70, 0·88		0·98	0·85, 1·12
	≥ 1/d		1·53	1·41, 1·67		1·17	1·05, 1·29		0·79	0·71, 0·89		0·98	0·85, 1·15
11 years		29 020			21 909			28 971			21 866		
	Never		–			–			–			–	
	< 1/week		1·10	0·97, 1·25		1·06	0·90, 1·23		0·85	0·73, 1·00		1·04	0·86, 1·26
	1–6/week		1·38	1·26, 1·51		1·19	1·06, 1·33		0·77	0·68, 0·88		0·90	0·77, 1·07
	≥ 1/d		1·69	1·54, 1·87		1·19	1·05, 1·35		0·77	0·68, 0·89		0·82	0·69, 0·98
14 years		28 804			21 594			28 746			21 550		
	Never		–			–			–			–	
	< 1/week		1·04	0·92, 1·19		0·99	0·84, 1·16		0·84	0·72, 0·99		1·06	0·87, 1·30
	1–6/week		1·34	1·22, 1·48		1·08	0·96, 1·22		0·78	0·69, 0·90		0·93	0·78, 1·10
	≥ 1/d		1·73	1·57, 1·91		1·16	1·03, 1·32		0·80	0·70, 0·92		0·91	0·76, 1·09
18 years		32 746			23 852			32 701			23 807		
	Never		–			–			–			–	
	< 1/week		1·09	0·95, 1·23		1·07	0·91, 1·25		0·65	0·56, 0·76		0·75	0·61, 0·91
	1–6/week		1·41	1·28, 1·55		1·13	1·00, 1·27		0·68	0·60, 0·77		0·79	0·67, 0·93
	≥ 1/d		1·86	1·69, 2·05		1·26	1·12, 1·42		0·69	0·60, 0·78		0·72	0·60, 0·86

ASB, artificially sweetened beverages; SSB, sugar-sweetened beverages; LGA, large for gestational age.

* OR (CI). ^†^Adjusted for maternal pre-pregnancy BMI, healthy eating index, age, smoking during pregnancy, physical activity in early pregnancy, duration of breastfeeding (except at birth), socio-economic status and paternal BMI.

Evaluating the difference in z-score at birth or BMI z-score dependent on maternal beverage consumption is shown; regarding ASB, the largest difference in BMI z-score was seen among the 18-year-olds whose mothers consumed ≥ 1 ASB/d compared with no consumption (BMI z-score 0·07 (95 % CI 0·03, 0·10)) (online Supplementary eTable S1). For SSB, the largest difference in BMI z-score was seen among the 18-year-olds whose mothers consumed ≥ 1 SSB/d compared with no consumption (BMI z-score −0·12 (95 % CI − 0·16, −0·07)) (online Supplementary eTable S1).

While we did not adjust for gestational weight gain in the primary analysis, doing so minimally attenuated the associations, and the associations persisted at ages 7, 11 and 18 years (online Supplementary eTable S3 and eTable S4). Similar results were seen when adjusting for total energy intake, where minimal changes were seen for ASB (online Supplementary eTable S5) and SSB; however, for SSB, the attenuation was more profound (online Supplementary eTable S6). When performing a substitution analysis substituting SSB with ASB and vice versa (see online Supplementary eTables S5 and S6), moderate attenuations were seen for SSB.

## Discussion

Our results indicate that daily ASB consumption compared with no ASB consumption during pregnancy is associated with a higher risk of overweight during childhood and adolescence, but not at birth or in infancy. We found a higher risk of being overweight at 7, 11 and 18 years among offspring whose mothers consumed 1–6 ASB/week or ≥ 1 ASB/d during pregnancy. Additionally, we found that consumption of ≥ 1 SSB/d during pregnancy was associated with a lower risk of overweight in the offspring at ages 11 and 18 years compared with no consumption. These results remained after adjustments for known risk factors for childhood obesity and overweight as well as in substitution models. Previous studies have found a higher risk of overweight during infancy in children exposed to the largest amounts of artificial sweeteners^(22,24)^. The incongruence with our results could be due to different populations and adjustment models. Notably, the observed effect sizes for these associations were modest, and reservations should be kept before basing clinical advice on these results.

We adjusted for a variety of covariates that are potential determinants of childhood obesity. We sought to account for lifestyle variables by adjusting for overall diet quality (healthy eating index), smoking in pregnancy, physical activity, duration of breastfeeding and SES. However, acknowledging that it is an inherent challenge to capture lifestyle factors in an observational study, residual confounding may persist. Our study focused on prenatal and early-life factors, and future investigations may benefit from considering ASB consumption during childhood and adolescence as a potential risk factor. Gestational weight gain was not included in the primary analysis, as we considered it a potential mediator of the association between ASB and childhood overweight, as a previous study showed an association between ASB consumption in pregnancy and higher gestational weight gain^(43)^.

Additionally, we found that daily consumption of SSB was associated with a trend towards a lower overweight risk in the offspring. Since the main difference between ASB and SSB is the sweetening agent, these findings may indicate that artificial sweeteners, rather than other components of the beverage, account for the association found for ASB. Still, differences between those choosing ASB over SSB remain; hence, residual confounding cannot be excluded. Additionally, we found a trend towards a decreased risk of overweight among children whose mothers reported daily consumption of SSB, seemingly suggesting a protective effect on childhood overweight. It should be noted, as shown in Table 1, that the group reporting daily consumption of ASB and the group reporting daily consumption of SSB differed on several baseline values, which could affect the results. For instance, women who reported daily consumption of SSB tended to have a higher SES, had a lower pre-pregnancy BMI and tended to breastfeed for a longer time compared with women who reported daily consumption of ASB. This indicates that the reference groups are differently composed, which could potentially introduce bias. This should be considered when interpreting results.

When comparing low-calorie beverages and calorie-dense beverages, it is likely to observe confounding due to differences in energy consumption. Indeed, as portrayed in Table 1, total energy intake in kcal was higher in the daily SSB-consumption group than in the daily ASB-consumption group. In a sub-analysis adjusting for total energy intake (kcal) instead of healthy eating index, the results for ASB were minimally attenuated, while for SSB, some regression towards the null was observed (online Supplementary eTable S5 and eTable S6). To further investigate if the substitution of ASB or SSB with other drinks would affect the results, we constructed an isocaloric substitution model. In this model, replacing ASB with SSB was associated with lower weight gain, which is consistent with findings from unadjusted analyses that consumption of ASB is associated with a higher risk of overweight.

The observed association between maternal ASB consumption and offspring overweight manifested in mid-childhood, while we saw no discernible correlation during infancy. There is a substantial time gap between the measurements at 12 months and 7 years, along with a large difference in overweight risk. Keeping in mind that rapid weight gain from 2 to 6 years of age increases the risk of manifest obesity^(44)^, longitudinal analyses are called for to fully comprehend the development of overweight in this age period concerning ASB consumption. In infancy, we saw a limited influence of ASB on overweight risk, which could indicate that changes appear when the offspring start choosing their own food to a larger extent. Furthermore, we found the highest OR for overweight among 18-year-olds exposed to ≥ 1 ASB/d during pregnancy. One possibility could be that dietary habits and lifestyle behaviour become increasingly independent of parental behaviours with increasing age^(45)^, while height has mostly stabilised at age 18^(46,47)^.

We found increasing BMI Z-score with increasing consumption of ASB, suggestive of a dose-dependent association between artificial sweetener consumption and risk for overweight in the offspring. One ASB or SSB was defined as 250 ml, corresponding to one regular glass of, for example, artificially sweetened soda. Danish Health Authorities recommend that adults consume no more than 500 ml of soda or non-carbonated sweetened beverages weekly^(48)^. According to our data, this recommendation was followed by 36 % of the women regarding SSB and 71 % of the women regarding ASB (Table 1). Our results highlight the importance of limiting consumption of SSB and ASB, and the low adherence to recommendations is concerning.

As previously described, some artificial sweeteners are transferred to the child via breast milk, enabling postnatal exposure. When the women report consuming artificial sweeteners during pregnancy, we speculate that these patterns continue postpartum, meaning that the children are further exposed to artificial sweeteners in infancy. Early exposure to artificial sweeteners showed contradictory evidence on metabolic effects in a systematic review^(49)^; however, no reports on infant artificial sweetener exposure were included. If the children, who were exposed to artificial sweeteners *in utero* continue exposure postnatally, this could account for some of the associations observed in our results. Studies on the consequences of infant exposure to artificial sweeteners are warranted to investigate this possible association.

Despite the robust sample size and extended follow-up, limitations inherent in observational studies, such as self-reported data and recall bias, remain in this study. Limitations include the reliance on self-reported beverage consumption, which holds uncertainty and makes it impossible to investigate specific artificial sweeteners. Furthermore, the questionnaire did not include other sources of artificial sweeteners, including tabletop sweeteners. Additionally, there have been substantial changes in artificial sweetener usage patterns since the data collection (1996–2003), with an increase in consumption^(50)^, which could impact the generalisability of our findings today. There is evidence of some selection bias in the DNBC, and despite inclusive inclusion criteria, some segments of the population may be underrepresented^(51)^.

Adjusting for a variety of confounders eliminated many potential participants from this study. Thus, the adjusted results are based on fewer participants than those available for the unadjusted analyses, resulting in less precise adjusted estimates. An influence by confounding factors cannot be discounted despite adjustment due to the uncontrolled environment intrinsic in observational studies. Characteristics for the participants at each follow-up are shown in online Supplementary eTable S2. When interpreting the results, it is important to consider that due to the extensive sample size, even minor associations may achieve statistical significance. Thus, the clinical implications of these associations may be modest.

Presently, women with diabetes and/or overweight are recommended to substitute SSB with artificially sweetened alternatives during pregnancy^(21)^. While this may be motivated by goals of limiting gestational weight gain^(52,53)^, this study suggests adverse long-term effects, as there is an observed tendency towards an increased risk of overweight in the offspring if these recommendations are met. We excluded women with diabetes in this analysis, but existing evidence shows an increased OR of overweight among children born to women who had gestational diabetes and were exposed to artificial sweeteners during pregnancy^(23)^.

In conclusion, we found in adjusted analyses that daily consumption of ASB during pregnancy increased the odds of overweight in mid-childhood and adolescence, but not in infancy. Furthermore, we found a trend suggesting that daily consumption of SSB during pregnancy was associated with a decreased risk of overweight in adolescence. Longitudinal evidence for the offspring at ages 2–6 years is warranted to better understand the association.

## Supporting information

Gjørup et al. supplementary material Gjørup et al. supplementary material

## Supplementary material

For supplementary material/s referred to in this article, please visit https://doi.org/10.1017/S0007114525000455.

## References

[1] Gulliford MC , Shibuya K & NCD Risk Factor Collaboration (2017) Worldwide trends in body-mass index, underweight, overweight, and obesity from 1975 to 2016: a pooled analysis of 2416 population-based measurement studies in 128·9 million children, adolescents, and adults. Lancet 390, 2627–2642. 29029897 10.1016/S0140-6736(17)32129-3 PMC5735219

[2] Powell-Wiley TM , Poirier P , Burke LE , et al. (2021) Obesity and cardiovascular disease: a scientific statement from the American Heart Association. Circulation 143, e984–e1010. 33882682 10.1161/CIR.0000000000000973 PMC8493650

[3] Maggio CA & Pi-Sunyer FX (2003) Obesity and type 2 diabetes. Endocrinol Metab Clin North Am 32, 805–822, viii. 14711063 10.1016/s0889-8529(03)00071-9

[4] Ng SW , Slining MM & Popkin BM (2012) Use of caloric and noncaloric sweeteners in US consumer packaged foods, 2005–2009. J Acad Nutr Diet 112, 1828–1834.e1821–1826. 23102182 10.1016/j.jand.2012.07.009 PMC3490437

[5] Godshall MA (2007) The expanding world of nutritive and non-nutritive sweeteners. Sugar J 69, 12–20.

[6] Chattopadhyay S , Raychaudhuri U & Chakraborty R (2014) Artificial sweeteners - a review. J Food Sci Technol 51, 611–621. 24741154 10.1007/s13197-011-0571-1 PMC3982014

[7] Sylvetsky AC , Jin Y , Clark EJ , et al. (2017) Consumption of low-calorie sweeteners among children and adults in the United States. J Acad Nutr Diet 117, 441–448.e442. 28087414 10.1016/j.jand.2016.11.004 PMC6176710

[8] Pearlman M , Obert J & Casey L (2017) The association between artificial sweeteners and obesity. Curr Gastroenterol Rep 19, 64. 29159583 10.1007/s11894-017-0602-9

[9] Organization GWH (2023) Use of Non-Sugar Sweeteners: WHO Guideline. Geneva: World Health Organization. 37256996

[10] Palmnäs MS , Cowan TE , Bomhof MR , et al. (2014) Low-dose aspartame consumption differentially affects gut microbiota–host metabolic interactions in the diet-induced obese rat. PLoS One 9, e109841. 25313461 10.1371/journal.pone.0109841 PMC4197030

[11] Suez J , Cohen Y , Valdés-Mas R , et al. (2022) Personalized microbiome-driven effects of non-nutritive sweeteners on human glucose tolerance. Cell 185, 3307–3328. 35987213 10.1016/j.cell.2022.07.016

[12] Suez J , Korem T , Zeevi D , et al. (2014) Artificial sweeteners induce glucose intolerance by altering the gut microbiota. Nature 514, 181–186. 25231862 10.1038/nature13793

[13] Weihrauch-Blüher S , Schwarz P & Klusmann JH (2019) Childhood obesity: increased risk for cardiometabolic disease and cancer in adulthood. Metabolism 92, 147–152. 30529454 10.1016/j.metabol.2018.12.001

[14] Robertson J , Schaufelberger M , Lindgren M , et al. (2019) Higher body mass index in adolescence predicts cardiomyopathy risk in midlife. Circulation 140, 117–125. 31132859 10.1161/CIRCULATIONAHA.118.039132 PMC6635044

[15] Qiao J , Dai LJ , Zhang Q , et al. (2020) A meta-analysis of the association between breastfeeding and early childhood obesity. J Pediatr Nurs 53, 57–66. 32464422 10.1016/j.pedn.2020.04.024

[16] Rayfield S & Plugge E (2017) Systematic review and meta-analysis of the association between maternal smoking in pregnancy and childhood overweight and obesity. J Epidemiol Community Health 71, 162–173. 27480843 10.1136/jech-2016-207376

[17] Sares-Jäske L , Grönqvist A , Mäki P , et al. (2022) Family socioeconomic status and childhood adiposity in Europe - a scoping review. Prev Med 160, 107095. 35594926 10.1016/j.ypmed.2022.107095

[18] Voerman E , Santos S , Patro Golab B , et al. (2019) Maternal body mass index, gestational weight gain, and the risk of overweight and obesity across childhood: an individual participant data meta-analysis. PLoS Med 16, e1002744. 30742624 10.1371/journal.pmed.1002744 PMC6370184

[19] Di Cesare M , Sorić M , Bovet P , et al. (2019) The epidemiological burden of obesity in childhood: a worldwide epidemic requiring urgent action. BMC Med 17, 212. 31760948 10.1186/s12916-019-1449-8 PMC6876113

[20] Smith JD , Fu E & Kobayashi MA (2020) Prevention and management of childhood obesity and its psychological and health comorbidities. Annu Rev Clin Psychol 16, 351–378. 32097572 10.1146/annurev-clinpsy-100219-060201 PMC7259820

[21] NHS (2019) Treatment Gestational Diabetes. https://www.nhs.uk/conditions/gestational-diabetes/treatment/ (accessed 07 January 2022).

[22] Plows JF , Aris IM , Rifas-Shiman SL , et al. (2022) Associations of maternal non-nutritive sweetener intake during pregnancy with offspring body mass index and body fat from birth to adolescence. Int J Obes (Lond) 46, 186–193. 34611285 10.1038/s41366-021-00897-0 PMC8784986

[23] Zhu Y , Olsen SF , Mendola P , et al. (2017) Maternal consumption of artificially sweetened beverages during pregnancy, and offspring growth through 7 years of age: a prospective cohort study. Int J Epidemiol 46, 1499–1508. 28586472 10.1093/ije/dyx095 PMC5837735

[24] Azad MB , Sharma AK , de Souza RJ , et al. (2016) Association between artificially sweetened beverage consumption during pregnancy and infant body mass index. JAMA Pediatr 170, 662–670. 27159792 10.1001/jamapediatrics.2016.0301

[25] Li G , Wang R , Zhang C , et al. (2022) Consumption of non-nutritive sweetener during pregnancy and weight gain in offspring: evidence from human studies. Nutrients 14, 5098. 36501127 10.3390/nu14235098 PMC9739060

[26] Gillman MW , Rifas-Shiman SL , Fernandez-Barres S , et al. (2017) Beverage intake during pregnancy and childhood adiposity. Pediatrics 140, e20170031. 28689188 10.1542/peds.2017-0031 PMC5527670

[27] von Poser Toigo E , Huffell AP , Mota CS , et al. (2015) Metabolic and feeding behavior alterations provoked by prenatal exposure to aspartame. Appetite 87, 168–174. 25543075 10.1016/j.appet.2014.12.213

[28] Sylvetsky AC , Conway EM , Malhotra S , et al. (2017) Development of sweet taste perception: implications for artificial sweetener use. Endocr Dev 32, 87–99. 28873386 10.1159/000475733

[29] Halasa BC , Sylvetsky AC , Conway EM , et al. (2021) Non-nutritive sweeteners in human amniotic fluid and cord blood: evidence of transplacental fetal exposure. Am J Perinatol 40, 1286–1291. 34500483 10.1055/s-0041-1735555

[30] Leth-Møller M , Duvald CS , Stampe S , et al. (2023) Transplacental transport of artificial sweeteners. Nutrients 15, 2063. 37432196 10.3390/nu15092063 PMC10181363

[31] Archibald AJ , Dolinsky VW & Azad MB (2018) Early-life exposure to non-nutritive sweeteners and the developmental origins of childhood obesity: global evidence from human and rodent studies. Nutrients 10, 194. 29439389 10.3390/nu10020194 PMC5852770

[32] Stampe S , Leth-Møller M , Greibe E , et al. (2022) Artificial sweeteners in breast milk: a clinical investigation with a kinetic perspective. Nutrients 14, 2635. 35807817 10.3390/nu14132635 PMC9268461

[33] Sylvetsky AC , Gardner AL , Bauman V , et al. (2015) Nonnutritive sweeteners in breast milk. J Toxicol Environ Health A 78, 1029–1032. 26267522 10.1080/15287394.2015.1053646 PMC5583633

[34] Olsen J , Melbye M , Olsen SF , et al. (2001) The Danish National Birth Cohort - its background, structure and aim. Scand J Public Health 29, 300–307. 11775787 10.1177/14034948010290040201

[35] Olsen SF , Mikkelsen TB , Knudsen VK , et al. (2007) Data collected on maternal dietary exposures in the Danish National Birth Cohort. Paediatr Perinat Epidemiol 21, 76–86. 17239183 10.1111/j.1365-3016.2007.00777.x

[36] Mikkelsen TB , Olsen SF , Rasmussen SE , et al. (2007) Relative validity of fruit and vegetable intake estimated by the food frequency questionnaire used in the Danish National Birth Cohort. Scand J Public Health 35, 172–179. 17454921 10.1080/14034940600975625

[37] Mikkelsen TB , Osler M & Olsen SF (2006) Validity of protein, retinol, folic acid and *n*-3 fatty acid intakes estimated from the food-frequency questionnaire used in the Danish National Birth Cohort. Public Health Nutr 9, 771–778. 16925883 10.1079/phn2005883

[38] Madsen MTB , Bjerregaard AA , Furtado JD , et al. (2019) Comparisons of estimated intakes and plasma concentrations of selected fatty acids in pregnancy. Nutrients 11, 568. 30845776 10.3390/nu11030568 PMC6470916

[39] Cole TJ , Bellizzi MC , Flegal KM , et al. (2000) Establishing a standard definition for child overweight and obesity worldwide: international survey. BMJ 320, 1240–1243. 10797032 10.1136/bmj.320.7244.1240 PMC27365

[40] Pedersen CB (2011) The Danish civil registration system. Scand J Public Health 39, 22–25. 21775345 10.1177/1403494810387965

[41] Bjerregaard AA , Halldorsson TI , Tetens I , et al. (2019) Mother’s dietary quality during pregnancy and offspring’s dietary quality in adolescence: follow-up from a national birth cohort study of 19 582 mother-offspring pairs. PLoS Med 16, e1002911. 31513597 10.1371/journal.pmed.1002911 PMC6742222

[42] Bech BH , Nohr EA , Vaeth M , et al. (2005) Coffee and fetal death: a cohort study with prospective data. Am J Epidemiol 162, 983–990. 16207803 10.1093/aje/kwi317

[43] Renault KM , Carlsen EM , Nørgaard K , et al. (2015) Intake of sweets, snacks and soft drinks predicts weight gain in obese pregnant women: detailed analysis of the results of a randomised controlled trial. PLoS One 10, e0133041. 26192183 10.1371/journal.pone.0133041 PMC4507874

[44] Geserick M , Vogel M , Gausche R , et al. (2018) Acceleration of BMI in early childhood and risk of sustained obesity. N Engl J Med 379, 1303–1312. 30281992 10.1056/NEJMoa1803527

[45] McKeown A & Nelson R (2018) Independent decision making of adolescents regarding food choice. Int J Consum Stud 42, 469–477.

[46] de Oliveira MH , Araújo J , Ramos E , et al. (2023) MULT: new height references and their efficiency in multi-ethnic populations. Am J Hum Biol 35, e23859. 36626316 10.1002/ajhb.23859

[47] Tinggaard J , Aksglaede L , Sørensen K , et al. (2014) The 2014 Danish references from birth to 20 years for height, weight and body mass index. Acta Paediatr 103, 214–224. 24127859 10.1111/apa.12468

[48] Authority DH (2024) Anbefalinger om kost (Dietary recommendations). https://sst.dk/da/Borger/En-sund-hverdag/Kost,-motion-og-hygiejne/Anbefalinger-om-kost (accessed 29 January 2024).

[49] Reid AE , Chauhan BF , Rabbani R , et al. (2016) Early exposure to nonnutritive sweeteners and long-term metabolic health: a systematic review. Pediatrics 137, e20153603. 26917671 10.1542/peds.2015-3603

[50] Sylvetsky AC , Figueroa J , Rother KI , et al. (2019) Trends in low-calorie sweetener consumption among pregnant women in the United States. Curr Dev Nutr 3, nzz004. 30931427 10.1093/cdn/nzz004 PMC6435448

[51] Jacobsen TN , Nohr EA & Frydenberg M (2010) Selection by socioeconomic factors into the Danish National Birth Cohort. Eur J Epidemiol 25, 349–355. 20349116 10.1007/s10654-010-9448-2

[52] Rasmussen KM , Catalano PM & Yaktine AL (2009) New guidelines for weight gain during pregnancy: what obstetrician/gynecologists should know. Curr Opin Obstet Gynecol 21, 521–526. 19809317 10.1097/GCO.0b013e328332d24e PMC2847829

[53] Johansson K , Bodnar LM , Stephansson O , et al. (2024) Safety of low weight gain or weight loss in pregnancies with class 1, 2, and 3 obesity: a population-based cohort study. Lancet 403, 1472–1481. 38555927 10.1016/S0140-6736(24)00255-1 PMC11097195

