# Supplementary material for: Consumption of artificial sweeteners during pregnancy and the risk of overweight in the offspring
Source: Br J Nutr. 2025 Mar 21;133(7):966–76. doi: 10.1017/S0007114525000455 (PMC12198349; doi:10.1017/S0007114525000455)
Supplement: Gjørup et al. supplementary material [file S0007114525000455sup001.docx]

Online only supplementary materials

Supplementary materials include:

[eTable S1: Difference in z-score at birth or body mass index z-score dependent on artificially sweetened and sugar-sweetened beverage consumption. 2](#_Toc187244618)

[eTable S2: Characteristics for the participants at each follow up. 4](#_Toc187244619)

[eTable S3: Odds ratio for large for gestational age birth weight or overweight at different ages dependent on maternal beverage consumption during pregnancy (+ adjustment for gestational weight gain). 6](#_Toc187244620)

[eTable S4: Difference in z-score at birth or body mass index z-score dependent on artificially sweetened and sugar-sweetened beverage consumption (+ adjustment for gestational weight gain). 8](#_Toc187244621)

[eTable S5: Odds ratio for large for gestational age birth weight or overweight at different ages dependent on maternal artificially sweetened beverage consumption in pregnancy + substitution model. 10](#_Toc187244622)

[eTable S6: Odds ratio for large for gestational age birth weight or overweight at different ages dependent on maternal sugar-sweetened beverage consumption in pregnancy + substitution model. 12](#_Toc187244623)

[eFigure S1: Timeline for the data collection in the DNBC. 14](#_Toc187244624)

[eFigure S2: Directed Acyclic Graph (DAG). 15](#_Toc187244625)

# eTable S1: Difference in z-score at birth or body mass index z-score dependent on artificially sweetened and sugar-sweetened beverage consumption.

|  | | **ASB consumption** | | | | **SSB consumption** | | | |
| --- | --- | --- | --- | --- | --- | --- | --- | --- | --- |
| **Age** | **Consumption**  **frequency** | **N** | **Unadjusted**^1^ | **N** | **Adjusted**^1,2^ | **N** | **Unadjusted**^1^ | **N** | **Adjusted**^1,2^ |
| Birth |  | 66,078 |  | 45,690 |  | 65,976 |  | 45,656 |  |
|  | never |  | Reference |  | — |  | — |  | — |
|  | <1/week |  | 0.00 (-0.02, 0.02) |  | -0.03 (-0.05, 0.00) |  | -0.01 (-0.04, 0.02) |  | 0.02 (-0.02, 0.05) |
|  | 1-6/week |  | 0.01 (-0.01, 0.03) |  | -0.03 (-0.05, -0.01) |  | 0.01 (-0.01, 0.04) |  | 0.05 (0.02, 0.08) |
|  | ≥1/day |  | 0.01 (-0.01, 0.03) |  | -0.02 (-0.04, 0.00) |  | 0.02 (-0.01, 0.04) |  | 0.09 (0.06, 0.12) |
| 5 months |  | 42,256 |  | 32,049 |  | 42,207 |  | 32,020 |  |
|  | never |  | — |  | — |  | — |  | — |
|  | <1/week |  | -0.03 (-0.06, 0.00) |  | -0.06 (-0.09, -0.02) |  | 0.00 (-0.04, 0.04) |  | 0.02 (-0.03, 0.07) |
|  | 1-6/week |  | 0.03 (0.01, 0.06) |  | -0.01 (-0.04, 0.02) |  | -0.02 (-0.06, 0.01) |  | 0.00 (-0.04, 0.04) |
|  | ≥1/day |  | 0.06 (0.03, 0.09) |  | 0.02 (-0.01, 0.05) |  | -0.02 (-0.06, 0.01) |  | 0.02 (-0.03, 0.06) |
| 12 months |  | 39,361 |  | 35,988 |  | 39,320 |  | 35,969 |  |
|  | never |  | — |  | — |  | — |  | — |
|  | <1/week |  | 0.01 (-0.02, 0.05) |  | -0.01 (-0.04, 0.03) |  | 0.01 (-0.04, 0.05) |  | 0.02 (-0.02, 0.07) |
|  | 1-6/week |  | 0.03 (0.01, 0.06) |  | 0.00 (-0.03, 0.02) |  | -0.02 (-0.06, 0.01) |  | 0.00 (-0.04, 0.04) |
|  | ≥1/day |  | 0.06 (0.04, 0.09) |  | 0.01 (-0.02, 0.04) |  | -0.03 (-0.07, 0.00) |  | 0.01 (-0.04, 0.05) |
| 7 years |  | 41,431 |  | 30,646 |  | 41,370 |  | 30,611 |  |
|  | never |  | — |  | — |  | — |  | — |
|  | <1/week |  | 0.06 (0.03, 0.09) |  | 0.03 (-0.01, 0.06) |  | -0.05 (-0.10, -0.01) |  | 0.02 (-0.03, 0.07) |
|  | 1-6/week |  | 0.10 (0.08, 0.13) |  | 0.04 (0.01, 0.07) |  | -0.09 (-0.13, -0.06) |  | -0.02 (-0.06, 0.02) |
|  | ≥1/day |  | 0.16 (0.14, 0.19) |  | 0.04 (0.01, 0.07) |  | -0.11 (-0.14, -0.07) |  | -0.02 (-0.06, 0.02) |
| 11 years |  | 29,020 |  | 21,909 |  | 28,971 |  | 21,866 |  |
|  | never |  | — |  | — |  | — |  | — |
|  | <1/week |  | 0.04 (0.00, 0.08) |  | 0.02 (-0.02, 0.06) |  | -0.08 (-0.13, -0.03) |  | 0.02 (-0.04, 0.07) |
|  | 1-6/week |  | 0.11 (0.08, 0.14) |  | 0.03 (-0.01, 0.06) |  | -0.12 (-0.16, -0.08) |  | -0.04 (-0.09, 0.01) |
|  | ≥1/day |  | 0.20 (0.16, 0.23) |  | 0.03 (0.00, 0.07) |  | -0.13 (-0.17, -0.08) |  | -0.06 (-0.11, -0.01) |
| 14 years |  | 28,804 |  | 21,594 |  | 28,746 |  | 21,550 |  |
|  | never |  | — |  | — |  | — |  | — |
|  | <1/week |  | 0.05 (0.01, 0.08) |  | 0.01 (-0.03, 0.05) |  | -0.07 (-0.12, -0.02) |  | 0.02 (-0.04, 0.07) |
|  | 1-6/week |  | 0.11 (0.08, 0.14) |  | 0.00 (-0.03, 0.04) |  | -0.10 (-0.14, -0.05) |  | -0.02 (-0.07, 0.03) |
|  | ≥1/day |  | 0.20 (0.17, 0.23) |  | 0.03 (-0.01, 0.06) |  | -0.09 (-0.14, -0.05) |  | -0.01 (-0.06, 0.04) |
| 18 years |  | 32,746 |  | 23,852 |  | 32,701 |  | 23,807 |  |
|  | never |  | — |  | — |  | — |  | — |
|  | <1/week |  | 0.08 (0.04, 0.11) |  | 0.04 (0.00, 0.07) |  | -0.15 (-0.20, -0.10) |  | -0.08 (-0.13, -0.03) |
|  | 1-6/week |  | 0.15 (0.13, 0.18) |  | 0.04 (0.01, 0.07) |  | -0.16 (-0.20, -0.12) |  | -0.09 (-0.14, -0.05) |
|  | ≥1/day |  | 0.25 (0.22, 0.28) |  | 0.07 (0.03, 0.10) |  | -0.17 (-0.21, -0.13) |  | -0.12 (-0.16, -0.07) |
| ^1^Values are mean difference in Z-score compared to no consumption (95%CI = 95%Confidence Interval). ^2^Adjusted for maternal pre-pregnancy body mass index, Healthy Eating Index, age, smoking during pregnancy, physical activity in early pregnancy, duration of breastfeeding (except at birth), socioeconomic status, and paternal body mass index.  ASB = artificially sweetened beverages. SSB = sugar-sweetened beverages. | | | | | | | | | |

# eTable S2: Characteristics for the participants at each follow up.

|  | **Total** | | **5 Months** | | **12 Months** | | **7 Years** | | **11 Years** | | **14 Years** | | **18 Years** | |
| --- | --- | --- | --- | --- | --- | --- | --- | --- | --- | --- | --- | --- | --- | --- |
| **Characteristic** | **N** | **N = 90,293**^1^ | **N** | **N = 55,576**^1^ | **N** | **N = 52,123**^1^ | **N** | **N = 51,444**^1^ | **N** | **N = 45,160**^1^ | **N** | **N = 34,902**^1^ | **N** | **N = 50,297**^1^ |
| Pre-pregnancy BMI | 83,195 | 23.5 (4.3) | 52,740 | 23.6 (4.2) | 49,522 | 23.6 (4.2) | 48,560 | 23.4 (4.0) | 42,257 | 23.2 (3.9) | 33,055 | 23.3 (4.0) | 46,855 | 23.5 (4.3) |
| Unknown |  | 7,098 |  | 2,836 |  | 2,601 |  | 2,884 |  | 2,903 |  | 1,847 |  | 3,442 |
| Smoking in pregnancy | 88,457 | 23,306 (26%) | 55,510 | 13,463 (24%) | 52,070 | 12,557 (24%) | 51,290 | 12,134 (24%) | 44,685 | 9,943 (22%) | 34,828 | 7,487 (21%) | 49,713 | 12,362 (25%) |
| Unknown |  | 1,836 |  | 66 |  | 53 |  | 154 |  | 475 |  | 74 |  | 584 |
| Socioeconomic status | 84,249 |  | 53,414 |  | 50,152 |  | 49,169 |  | 42,788 |  | 33,436 |  | 47,441 |  |
| Leaders or long/medium long education |  | 56,469 (67%) |  | 36,441 (68%) |  | 34,141 (68%) |  | 34,302 (70%) |  | 30,840 (72%) |  | 24,196 (72%) |  | 32,772 (69%) |
| Unskilled, other work or receives public benefits |  | 3,331 (4.0%) |  | 1,714 (3.2%) |  | 1,614 (3.2%) |  | 1,553 (3.2%) |  | 1,118 (2.6%) |  | 856 (2.6%) |  | 1,593 (3.4%) |
| Working class, craftsmen, short education or under education |  | 24,449 (29%) |  | 15,259 (29%) |  | 14,397 (29%) |  | 13,314 (27%) |  | 10,830 (25%) |  | 8,384 (25%) |  | 13,076 (28%) |
| Unknown |  | 6,044 |  | 2,162 |  | 1,971 |  | 2,275 |  | 2,372 |  | 1,466 |  | 2,856 |
| Maternal SSB intake | 67,032 |  | 44,248 |  | 41,650 |  | 42,618 |  | 37,449 |  | 29,126 |  | 39,035 |  |
| <500ml/week |  | 23,833 (36%) |  | 15,811 (36%) |  | 14,959 (36%) |  | 15,263 (36%) |  | 13,258 (35%) |  | 10,462 (36%) |  | 13,803 (35%) |
| >= 1500 ml/week |  | 24,864 (37%) |  | 16,331 (37%) |  | 15,321 (37%) |  | 15,662 (37%) |  | 13,749 (37%) |  | 10,497 (36%) |  | 14,382 (37%) |
| 500-1500 ml/week |  | 18,335 (27%) |  | 12,106 (27%) |  | 11,370 (27%) |  | 11,693 (27%) |  | 10,442 (28%) |  | 8,167 (28%) |  | 10,850 (28%) |
| Unknown |  | 23,261 |  | 11,328 |  | 10,473 |  | 8,826 |  | 7,711 |  | 5,776 |  | 11,262 |
| Maternal ASB intake | 67,005 |  | 44,239 |  | 41,641 |  | 42,601 |  | 37,451 |  | 29,141 |  | 39,025 |  |
| <500ml/week |  | 47,644 (71%) |  | 31,399 (71%) |  | 29,437 (71%) |  | 30,689 (72%) |  | 27,292 (73%) |  | 21,215 (73%) |  | 27,971 (72%) |
| >= 1500 ml/week |  | 11,776 (18%) |  | 7,806 (18%) |  | 7,437 (18%) |  | 7,220 (17%) |  | 6,112 (16%) |  | 4,751 (16%) |  | 6,715 (17%) |
| 500-1500 ml/week |  | 7,585 (11%) |  | 5,034 (11%) |  | 4,767 (11%) |  | 4,692 (11%) |  | 4,047 (11%) |  | 3,175 (11%) |  | 4,339 (11%) |
| Unknown |  | 23,288 |  | 11,337 |  | 10,482 |  | 8,843 |  | 7,709 |  | 5,761 |  | 11,272 |
| Mean age, months |  |  | 54,314 | 5.28 (1.00) | 50,497 | 12.45 (1.07) |  |  |  |  |  |  |  |  |
| Unknown |  |  |  | 1,262 |  | 1,626 |  |  |  |  |  |  |  |  |
| Mean age, years |  |  |  |  |  |  | 50,960 | 7.04 (0.34) | 45,160 | 11.42 (0.63) | 34,621 | 14.16 (0.14) | 50,297 | 18.35 (0.16) |
| Unknown |  |  |  |  |  |  |  | 484 |  | 0 |  | 281 |  | 0 |
| ^1^Mean (SD); n (%). BMI = body mass index.  ASB = artificially sweetened beverages. SSB = sugar-sweetened beverages. | | | | | | | | | | | | | | |

# eTable S3: Odds ratio for large for gestational age birth weight or overweight at different ages dependent on maternal beverage consumption during pregnancy (+ adjustment for gestational weight gain).

|  | | **ASB consumption** | | | | **SSB consumption** | | | |
| --- | --- | --- | --- | --- | --- | --- | --- | --- | --- |
| **Age** | **Consumption**  **frequency** | **N** | **Unadjusted** | **N** | **Adjusted**^2^ | **N** | **Unadjusted** | **N** | **Adjusted**^2^ |
|  |  |  | **OR (95%CI)**^1^ |  | **OR (95%CI)**^1^ |  | **OR (95%CI)**^1^ |  | **OR (95%CI)**^1^ |
| LGA at birth |  | 66,078 |  | 37,920 |  | 65,976 |  | 37,894 |  |
|  | never |  | — |  | — |  | — |  | — |
|  | <1/week |  | 0.92 (0.73, 1.13) |  | 0.81 (0.60, 1.07) |  | 0.86 (0.65, 1.13) |  | 0.96 (0.67, 1.39) |
|  | 1-6/week |  | 1.12 (0.96, 1.31) |  | 0.99 (0.81, 1.21) |  | 0.94 (0.76, 1.18) |  | 1.21 (0.90, 1.66) |
|  | ≥1/day |  | 1.08 (0.91, 1.28) |  | 0.81 (0.64, 1.02) |  | 1.01 (0.81, 1.28) |  | 1.29 (0.94, 1.79) |
| 5 months |  | 42,256 |  | 31,820 |  | 42,207 |  | 31,791 |  |
|  | never |  | — |  | — |  | — |  | — |
|  | <1/week |  | 0.85 (0.77, 0.95) |  | 0.82 (0.72, 0.93) |  | 0.96 (0.84, 1.09) |  | 0.99 (0.85, 1.16) |
|  | 1-6/week |  | 1.05 (0.97, 1.14) |  | 0.99 (0.90, 1.09) |  | 0.86 (0.77, 0.97) |  | 0.89 (0.78, 1.01) |
|  | ≥1/day |  | 1.05 (0.97, 1.15) |  | 0.97 (0.87, 1.07) |  | 0.92 (0.83, 1.04) |  | 0.97 (0.85, 1.12) |
| 12 months |  | 39,361 |  | 30,147 |  | 39,320 |  | 30,129 |  |
|  | never |  | — |  | — |  | — |  | — |
|  | <1/week |  | 0.94 (0.84, 1.05) |  | 0.87 (0.76, 0.98) |  | 0.94 (0.82, 1.07) |  | 1.02 (0.87, 1.20) |
|  | 1-6/week |  | 1.08 (1.00, 1.18) |  | 0.99 (0.90, 1.09) |  | 0.89 (0.79, 1.00) |  | 0.99 (0.87, 1.14) |
|  | ≥1/day |  | 1.07 (0.98, 1.17) |  | 0.95 (0.85, 1.05) |  | 0.90 (0.81, 1.02) |  | 1.05 (0.91, 1.22) |
| 7 years |  | 41,431 |  | 25,870 |  | 41,370 |  | 25,838 |  |
|  | never |  | — |  | — |  | — |  | — |
|  | <1/week |  | 1.10 (0.98, 1.22) |  | 1.00 (0.86, 1.16) |  | 0.84 (0.73, 0.96) |  | 1.04 (0.87, 1.25) |
|  | 1-6/week |  | 1.34 (1.23, 1.45) |  | 1.21 (1.09, 1.35) |  | 0.79 (0.70, 0.88) |  | 0.98 (0.84, 1.15) |
|  | ≥1/day |  | 1.53 (1.41, 1.67) |  | 1.12 (0.99, 1.25) |  | 0.79 (0.71, 0.89) |  | 0.95 (0.81, 1.13) |
| 11 years |  | 29,020 |  | 19,095 |  | 28,971 |  | 19,053 |  |
|  | never |  | — |  | — |  | — |  | — |
|  | <1/week |  | 1.10 (0.97, 1.25) |  | 1.07 (0.90, 1.26) |  | 0.85 (0.73, 1.00) |  | 1.04 (0.85, 1.27) |
|  | 1-6/week |  | 1.38 (1.26, 1.51) |  | 1.17 (1.03, 1.32) |  | 0.77 (0.68, 0.88) |  | 0.92 (0.77, 1.10) |
|  | ≥1/day |  | 1.69 (1.54, 1.87) |  | 1.17 (1.02, 1.33) |  | 0.77 (0.68, 0.89) |  | 0.82 (0.68, 0.99) |
| 14 years |  | 28,804 |  | 18,771 |  | 28,746 |  | 18,724 |  |
|  | never |  | — |  | — |  | — |  | — |
|  | <1/week |  | 1.04 (0.92, 1.19) |  | 1.01 (0.85, 1.19) |  | 0.84 (0.72, 0.99) |  | 1.10 (0.89, 1.36) |
|  | 1-6/week |  | 1.34 (1.22, 1.48) |  | 1.04 (0.92, 1.18) |  | 0.78 (0.69, 0.90) |  | 0.99 (0.82, 1.20) |
|  | ≥1/day |  | 1.73 (1.57, 1.91) |  | 1.10 (0.96, 1.25) |  | 0.80 (0.70, 0.92) |  | 0.98 (0.80, 1.19) |
| 18 years |  | 32,746 |  | 19,973 |  | 32,701 |  | 19,935 |  |
|  | never |  | — |  | — |  | — |  | — |
|  | <1/week |  | 1.09 (0.95, 1.23) |  | 1.08 (0.90, 1.28) |  | 0.65 (0.56, 0.76) |  | 0.75 (0.60, 0.93) |
|  | 1-6/week |  | 1.41 (1.28, 1.55) |  | 1.19 (1.04, 1.35) |  | 0.68 (0.60, 0.77) |  | 0.78 (0.65, 0.93) |
|  | ≥1/day |  | 1.86 (1.69, 2.05) |  | 1.28 (1.12, 1.46) |  | 0.69 (0.60, 0.78) |  | 0.70 (0.58, 0.85) |
| ^1^OR = Odds Ratio (95%CI = 95%Confidence Interval). ^2^Adjusted for maternal pre-pregnancy BMI, gestational weight gain, Healthy Eating Index, age, smoking during pregnancy, physical activity in early pregnancy, duration of breastfeeding (except at birth), socioeconomic status, and paternal BMI.  LGA = large for gestational age. ASB = artificially sweetened beverages. SSB = sugar-sweetened beverages. | | | | | | | | | |

# eTable S4: Difference in z-score at birth or body mass index z-score dependent on artificially sweetened and sugar-sweetened beverage consumption (+ adjustment for gestational weight gain).

|  | | **ASB consumption** | | | | **SSB consumption** | | | |
| --- | --- | --- | --- | --- | --- | --- | --- | --- | --- |
| Age | **Consumption**  **frequency** | **N** | **Unadjusted**^1^ | **N** | **Adjusted**^1,2^ | **N** | **Unadjusted**^1^ | **N** | **Adjusted**^1,2^ |
| Birth |  | 66,078 |  | 37,920 |  | 65,976 |  | 37,894 |  |
|  | never |  | — |  | — |  | — |  | — |
|  | <1/week |  | 0.00 (-0.02, 0.02) |  | -0.02 (-0.05, 0.00) |  | -0.01 (-0.04, 0.02) |  | 0.03 (-0.01, 0.07) |
|  | 1-6/week |  | 0.01 (-0.01, 0.03) |  | -0.04 (-0.06, -0.02) |  | 0.01 (-0.01, 0.04) |  | 0.07 (0.03, 0.10) |
|  | ≥1/day |  | 0.01 (-0.01, 0.03) |  | -0.04 (-0.07, -0.02) |  | 0.02 (-0.01, 0.04) |  | 0.11 (0.07, 0.14) |
| 5 months |  | 42,256 |  | 31,820 |  | 42,207 |  | 31,791 |  |
|  | never |  | — |  | — |  | — |  | — |
|  | <1/week |  | -0.03 (-0.06, 0.00) |  | -0.05 (-0.09, -0.02) |  | 0.00 (-0.04, 0.04) |  | 0.02 (-0.03, 0.07) |
|  | 1-6/week |  | 0.03 (0.01, 0.06) |  | -0.01 (-0.04, 0.02) |  | -0.02 (-0.06, 0.01) |  | 0.00 (-0.04, 0.04) |
|  | ≥1/day |  | 0.06 (0.03, 0.09) |  | 0.01 (-0.02, 0.04) |  | -0.02 (-0.06, 0.01) |  | 0.02 (-0.03, 0.06) |
| 12 months |  | 39,361 |  | 30,147 |  | 39,320 |  | 30,129 |  |
|  | never |  | — |  | — |  | — |  | — |
|  | <1/week |  | 0.01 (-0.02, 0.05) |  | -0.01 (-0.04, 0.03) |  | 0.01 (-0.04, 0.05) |  | 0.03 (-0.02, 0.08) |
|  | 1-6/week |  | 0.03 (0.01, 0.06) |  | -0.01 (-0.04, 0.02) |  | -0.02 (-0.06, 0.01) |  | 0.01 (-0.04, 0.05) |
|  | ≥1/day |  | 0.06 (0.04, 0.09) |  | 0.00 (-0.03, 0.03) |  | -0.03 (-0.07, 0.00) |  | 0.01 (-0.03, 0.06) |
| 7 years |  | 41,431 |  | 25,870 |  | 41,370 |  | 25,838 |  |
|  | never |  | — |  | — |  | — |  | — |
|  | <1/week |  | 0.06 (0.03, 0.09) |  | 0.03 (-0.01, 0.07) |  | -0.05 (-0.10, -0.01) |  | 0.02 (-0.03, 0.07) |
|  | 1-6/week |  | 0.10 (0.08, 0.13) |  | 0.03 (0.00, 0.07) |  | -0.09 (-0.13, -0.06) |  | -0.03 (-0.08, 0.01) |
|  | ≥1/day |  | 0.16 (0.14, 0.19) |  | 0.03 (-0.01, 0.06) |  | -0.11 (-0.14, -0.07) |  | -0.03 (-0.08, 0.01) |
| 11 years |  | 29,020 |  | 19,095 |  | 28,971 |  | 19,053 |  |
|  | never |  | — |  | — |  | — |  | — |
|  | <1/week |  | 0.04 (0.00, 0.08) |  | 0.02 (-0.03, 0.06) |  | -0.08 (-0.13, -0.03) |  | 0.02 (-0.03, 0.08) |
|  | 1-6/week |  | 0.11 (0.08, 0.14) |  | 0.02 (-0.01, 0.05) |  | -0.12 (-0.16, -0.08) |  | -0.03 (-0.08, 0.02) |
|  | ≥1/day |  | 0.20 (0.16, 0.23) |  | 0.03 (0.00, 0.07) |  | -0.13 (-0.17, -0.08) |  | -0.05 (-0.11, 0.00) |
| 14 years |  | 28,804 |  | 18,771 |  | 28,746 |  | 18,724 |  |
|  | never |  | — |  | — |  | — |  | — |
|  | <1/week |  | 0.05 (0.01, 0.08) |  | 0.02 (-0.02, 0.06) |  | -0.07 (-0.12, -0.02) |  | 0.02 (-0.04, 0.08) |
|  | 1-6/week |  | 0.11 (0.08, 0.14) |  | 0.00 (-0.03, 0.04) |  | -0.10 (-0.14, -0.05) |  | -0.01 (-0.07, 0.04) |
|  | ≥1/day |  | 0.20 (0.17, 0.23) |  | 0.02 (-0.02, 0.06) |  | -0.09 (-0.14, -0.05) |  | -0.01 (-0.06, 0.05) |
| 18 years |  | 32,746 |  | 19,973 |  | 32,701 |  | 19,935 |  |
|  | never |  | — |  | — |  | — |  | — |
|  | <1/week |  | 0.08 (0.04, 0.11) |  | 0.04 (0.00, 0.08) |  | -0.15 (-0.20, -0.10) |  | -0.08 (-0.14, -0.02) |
|  | 1-6/week |  | 0.15 (0.13, 0.18) |  | 0.05 (0.01, 0.08) |  | -0.16 (-0.20, -0.12) |  | -0.10 (-0.14, -0.05) |
|  | ≥1/day |  | 0.25 (0.22, 0.28) |  | 0.06 (0.03, 0.10) |  | -0.17 (-0.21, -0.13) |  | -0.12 (-0.17, -0.07) |
| ^1^Values are mean difference in Z-score compared to no consumption (95%CI = 95%Confidence Interval). ^2^Adjusted for maternal pre-pregnancy body mass index, gestational weight gain, Healthy Eating Index, age, smoking during pregnancy, physical activity in early pregnancy, duration of breastfeeding (except at birth), socioeconomic status, and paternal body mass index.  ASB = artificially sweetened beverages. SSB = sugar-sweetened beverages. | | | | | | | | | |

# eTable S5: Odds ratio for large for gestational age birth weight or overweight at different ages dependent on maternal artificially sweetened beverage consumption in pregnancy + substitution model.

| **Age** | **Consumption frequency (ASB)** | **N** | **Unadjusted** | **N** | **Original analysis**^2^ | **N** | **Adjusted for TEI**^3^ | **N** | **Substitution**^4^ |
| --- | --- | --- | --- | --- | --- | --- | --- | --- | --- |
|  |  |  | **OR (95%CI)**^1^ |  | **OR (95%CI)**^1^ |  | **OR (95%CI)**^1^ |  | **OR (95%CI)**^1^ |
| LGA at birth |  | 66,078 |  | 45,690 |  | 44,074 |  | 43,226 |  |
|  | never |  | — |  | — |  | — |  | — |
|  | <1/week |  | 0.92 (0.73, 1.13) |  | 0.79 (0.60, 1.02) |  | 0.81 (0.61 to 1.05) |  | 0.86 (0.65 to 1.12) |
|  | 1-6/week |  | 1.12 (0.96, 1.31) |  | 0.99 (0.82, 1.19) |  | 0.99 (0.82 to 1.20) |  | 1.03 (0.85 to 1.25) |
|  | ≥1/day |  | 1.08 (0.91, 1.28) |  | 0.89 (0.73, 1.10) |  | 0.89 (0.72 to 1.09) |  | 0.94 (0.76 to 1.17) |
| 5 months |  | 42,256 |  | 32,049 |  | 30,955 |  | 30,399 |  |
|  | never |  | — |  | — |  | — |  | — |
|  | <1/week |  | 0.85 (0.77, 0.95) |  | 0.81 (0.71, 0.92) |  | 0.83 (0.72 to 0.94) |  | 0.81 (0.71 to 0.93) |
|  | 1-6/week |  | 1.05 (0.97, 1.14) |  | 1.00 (0.91, 1.10) |  | 1.02 (0.93 to 1.12) |  | 1.01 (0.92 to 1.11) |
|  | ≥1/day |  | 1.05 (0.97, 1.15) |  | 0.98 (0.88, 1.08) |  | 0.97 (0.88 to 1.08) |  | 0.96 (0.86 to 1.06) |
| 12 months |  | 39,361 |  | 35,988 |  | 34,671 |  | 34,044 |  |
|  | never |  | — |  | — |  | — |  | — |
|  | <1/week |  | 0.94 (0.84, 1.05) |  | 0.90 (0.80, 1.01) |  | 0.90 (0.79 to 1.01) |  | 0.89 (0.79 to 1.01) |
|  | 1-6/week |  | 1.08 (1.00, 1.18) |  | 1.00 (0.92, 1.09) |  | 1.00 (0.92 to 1.10) |  | 0.99 (0.90 to 1.08) |
|  | ≥1/day |  | 1.07 (0.98, 1.17) |  | 0.96 (0.87, 1.05) |  | 0.95 (0.87 to 1.05) |  | 0.94 (0.85 to 1.04) |
| 7 years |  | 41,431 |  | 30,646 |  | 29,766 |  | 29,226 |  |
|  | never |  | — |  | — |  | — |  | — |
|  | <1/week |  | 1.10 (0.98, 1.22) |  | 1.01 (0.88, 1.15) |  | 1.02 (0.89 to 1.17) |  | 1.00 (0.87 to 1.15) |
|  | 1-6/week |  | 1.34 (1.23, 1.45) |  | 1.19 (1.08, 1.32) |  | 1.18 (1.07 to 1.31) |  | 1.18 (1.06 to 1.31) |
|  | ≥1/day |  | 1.53 (1.41, 1.67) |  | 1.17 (1.05, 1.29) |  | 1.16 (1.04 to 1.29) |  | 1.16 (1.03 to 1.29) |
| 11 years |  | 29,020 |  | 21,909 |  | 21,787 |  | 21,392 |  |
|  | never |  | — |  | — |  | — |  | — |
|  | <1/week |  | 1.10 (0.97, 1.25) |  | 1.06 (0.90, 1.23) |  | 1.04 (0.89 to 1.22) |  | 1.03 (0.88 to 1.21) |
|  | 1-6/week |  | 1.38 (1.26, 1.51) |  | 1.19 (1.06, 1.33) |  | 1.20 (1.07 to 1.34) |  | 1.19 (1.05 to 1.33) |
|  | ≥1/day |  | 1.69 (1.54, 1.87) |  | 1.19 (1.05, 1.35) |  | 1.18 (1.05 to 1.34) |  | 1.15 (1.01 to 1.31) |
| 14 years |  | 28,804 |  | 21,594 |  | 21,404 |  | 21,023 |  |
|  | never |  | — |  | — |  | — |  | — |
|  | <1/week |  | 1.04 (0.92, 1.19) |  | 0.99 (0.84, 1.16) |  | 0.98 (0.83 to 1.14) |  | 0.97 (0.83 to 1.14) |
|  | 1-6/week |  | 1.34 (1.22, 1.48) |  | 1.08 (0.96, 1.22) |  | 1.08 (0.96 to 1.22) |  | 1.07 (0.94 to 1.21) |
|  | ≥1/day |  | 1.73 (1.57, 1.91) |  | 1.16 (1.03, 1.32) |  | 1.16 (1.03 to 1.32) |  | 1.17 (1.02 to 1.33) |
| 18 years |  | 32,746 |  | 23,852 |  | 22,967 |  | 22,546 |  |
|  | never |  | — |  | — |  | — |  | — |
|  | <1/week |  | 1.09 (0.95, 1.23) |  | 1.07 (0.91, 1.25) |  | 1.04 (0.88 to 1.22) |  | 1.03 (0.87 to 1.22) |
|  | 1-6/week |  | 1.41 (1.28, 1.55) |  | 1.13 (1.00, 1.27) |  | 1.12 (0.99 to 1.27) |  | 1.11 (0.98 to 1.26) |
|  | ≥1/day |  | 1.86 (1.69, 2.05) |  | 1.26 (1.12, 1.42) |  | 1.24 (1.10 to 1.41) |  | 1.23 (1.08 to 1.40) |
| ^1^OR = Odds Ratio (CI = Confidence Interval).  ^2^Adjusted for maternal pre-pregnancy body mass index, Healthy Eating Index, age, smoking during pregnancy, physical activity in early pregnancy, duration of breastfeeding (except at birth), socioeconomic status, and paternal body mass index.  ^3^Adjusted for maternal pre-pregnancy body mass index, Total Energy Intake, age, smoking during pregnancy, physical activity in early pregnancy, duration of breastfeeding (except at birth), socioeconomic status, and paternal body mass index.  ^4^Adjusted for the same covariates as (3) + adjustment for maternal consumption of sugar-sweetened beverages. ASB = artificially sweetened beverages. SSB = sugar-sweetened beverages. LGA = large for gestational age. TEI = Total energy intake. | | | | | | | | | |

# eTable S6: Odds ratio for large for gestational age birth weight or overweight at different ages dependent on maternal sugar-sweetened beverage consumption in pregnancy + substitution model.

| **Age** | **Consumption frequency (SSB)** | **N** | **Unadjusted** | **N** | **Original analysis**^2^ | **N** | **Adjusted for TEI**^3^ | **N** | **Substitution**^4^ |
| --- | --- | --- | --- | --- | --- | --- | --- | --- | --- |
|  |  |  | **OR (95%CI)**^1^ |  | **OR (95%CI)**^1^ |  | **OR (95%CI)**^1^ |  | **OR (95%CI)**^1^ |
| LGA at birth |  | 65,976 |  | 45,656 |  | 44,031 |  | 43,226 |  |
|  | never |  | — |  | — |  | — |  | — |
|  | <1/week |  | 0.86 (0.65, 1.13) |  | 0.90 (0.65, 1.24) |  | 0.87 (0.63 to 1.21) |  | 0.86 (0.62 to 1.20) |
|  | 1-6/week |  | 0.94 (0.76, 1.18) |  | 1.03 (0.79, 1.35) |  | 0.97 (0.75 to 1.28) |  | 0.96 (0.73 to 1.27) |
|  | ≥1/day |  | 1.01 (0.81, 1.28) |  | 1.15 (0.87, 1.53) |  | 1.01 (0.77 to 1.34) |  | 1.00 (0.75 to 1.34) |
| 5 months |  | 42,207 |  | 32,020 |  | 30,925 |  | 30,399 |  |
|  | never |  | — |  | — |  | — |  | — |
|  | <1/week |  | 0.96 (0.84, 1.09) |  | 0.99 (0.85, 1.16) |  | 0.98 (0.84 to 1.14) |  | 0.99 (0.84 to 1.16) |
|  | 1-6/week |  | 0.86 (0.77, 0.97) |  | 0.89 (0.78, 1.02) |  | 0.85 (0.75 to 0.97) |  | 0.85 (0.74 to 0.97) |
|  | ≥1/day |  | 0.92 (0.83, 1.04) |  | 0.98 (0.85, 1.13) |  | 0.91 (0.79 to 1.04) |  | 0.90 (0.79 to 1.04) |
| 12 months |  | 39,320 |  | 35,969 |  | 34,650 |  | 34,044 |  |
|  | never |  | — |  | — |  | — |  | — |
|  | <1/week |  | 0.94 (0.82, 1.07) |  | 0.97 (0.84, 1.13) |  | 0.99 (0.86 to 1.15) |  | 1.00 (0.86 to 1.17) |
|  | 1-6/week |  | 0.89 (0.79, 1.00) |  | 0.93 (0.82, 1.05) |  | 0.93 (0.82 to 1.05) |  | 0.93 (0.82 to 1.06) |
|  | ≥1/day |  | 0.90 (0.81, 1.02) |  | 0.99 (0.87, 1.12) |  | 0.97 (0.85 to 1.10) |  | 0.96 (0.84 to 1.10) |
| 7 years |  | 41,370 |  | 30,611 |  | 29,723 |  | 29,226 |  |
|  | never |  | — |  | — |  | — |  | — |
|  | <1/week |  | 0.84 (0.73, 0.96) |  | 1.04 (0.89, 1.23) |  | 1.03 (0.87 to 1.22) |  | 1.06 (0.90 to 1.26) |
|  | 1-6/week |  | 0.79 (0.70, 0.88) |  | 0.98 (0.85, 1.12) |  | 0.97 (0.84 to 1.11) |  | 1.0 (0.86 to 1.15) |
|  | ≥1/day |  | 0.79 (0.71, 0.89) |  | 0.98 (0.85, 1.15) |  | 0.95 (0.82 to 1.10) |  | 0.99 (0.85 to 1.16) |
| 11 years |  | 28,971 |  | 21,866 |  | 21,733 |  | 21,392 |  |
|  | never |  | — |  | — |  | — |  | — |
|  | <1/week |  | 0.85 (0.73, 1.00) |  | 1.04 (0.86, 1.26) |  | 1.05 (0.87 to 1.27) |  | 1.08 (0.89 to 1.32) |
|  | 1-6/week |  | 0.77 (0.68, 0.88) |  | 0.90 (0.77, 1.07) |  | 0.92 (0.79 to 1.09) |  | 0.96 (0.81 to 1.13) |
|  | ≥1/day |  | 0.77 (0.68, 0.89) |  | 0.82 (0.69, 0.98) |  | 0.82 (0.69 to 0.98) |  | 0.87 (0.73 to 1.04) |
| 14 years |  | 28,746 |  | 21,550 |  | 21,348 |  | 21,023 |  |
|  | never |  | — |  | — |  | — |  | — |
|  | <1/week |  | 0.84 (0.72, 0.99) |  | 1.06 (0.87, 1.30) |  | 1.08 (0.88 to 1.31) |  | 1.12 (0.91 to 1.37) |
|  | 1-6/week |  | 0.78 (0.69, 0.90) |  | 0.93 (0.78, 1.10) |  | 0.95 (0.80 to 1.13) |  | 0.99 (0.83 to 1.18) |
|  | ≥1/day |  | 0.80 (0.70, 0.92) |  | 0.91 (0.76, 1.09) |  | 0.94 (0.79 to 1.12) |  | 1.00 (0.83 to 1.21) |
| 18 years |  | 32,701 |  | 23,807 |  | 22,923 |  | 22,546 |  |
|  | never |  | — |  | — |  | — |  | — |
|  | <1/week |  | 0.65 (0.56, 0.76) |  | 0.75 (0.61, 0.91) |  | 0.76 (0.62 to 0.93) |  | 0.79 (0.64 to 0.97) |
|  | 1-6/week |  | 0.68 (0.60, 0.77) |  | 0.79 (0.67, 0.93) |  | 0.83 (0.71 to 0.98) |  | 0.89 (0.75 to 1.06) |
|  | ≥1/day |  | 0.69 (0.60, 0.78) |  | 0.72 (0.60, 0.86) |  | 0.78 (0.66 to 0.93) |  | 0.85 (0.71 to 1.02) |
| ^1^OR = Odds Ratio (CI = Confidence Interval).  ^2^Adjusted for maternal pre-pregnancy body mass index, Healthy Eating Index, age, smoking during pregnancy, physical activity in early pregnancy, duration of breastfeeding (except at birth), socioeconomic status, and paternal body mass index.  ^3^Adjusted for maternal pre-pregnancy body mass index, Total Energy Intake, age, smoking during pregnancy, physical activity in early pregnancy, duration of breastfeeding (except at birth), socioeconomic status, and paternal body mass index.  ^4^Adjusted for the same covariates as (3) + adjustment for maternal consumption of artificially sweetened beverages. ASB = artificially sweetened beverages. SSB = sugar-sweetened beverages. LGA = large for gestational age. TEI = Total energy intake. | | | | | | | | | |

# eFigure S1: Timeline for the data collection in the DNBC.


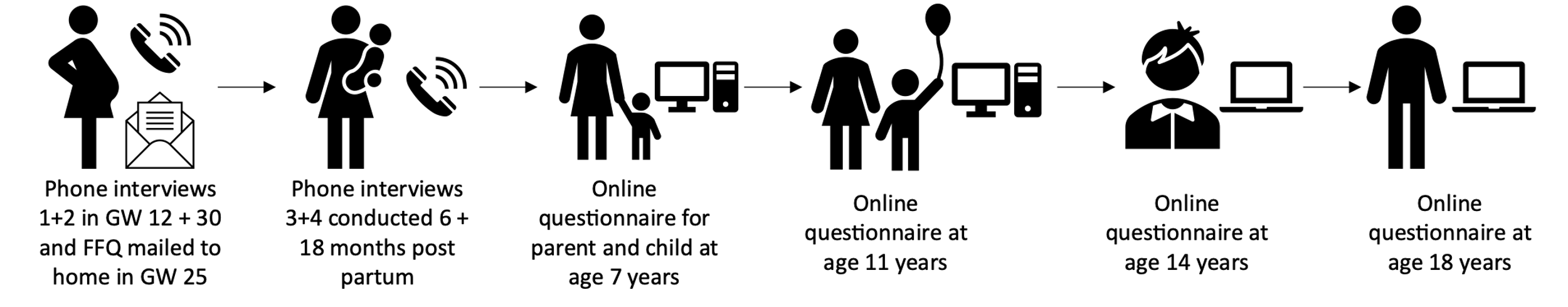


# eFigure S2: Directed Acyclic Graph (DAG).


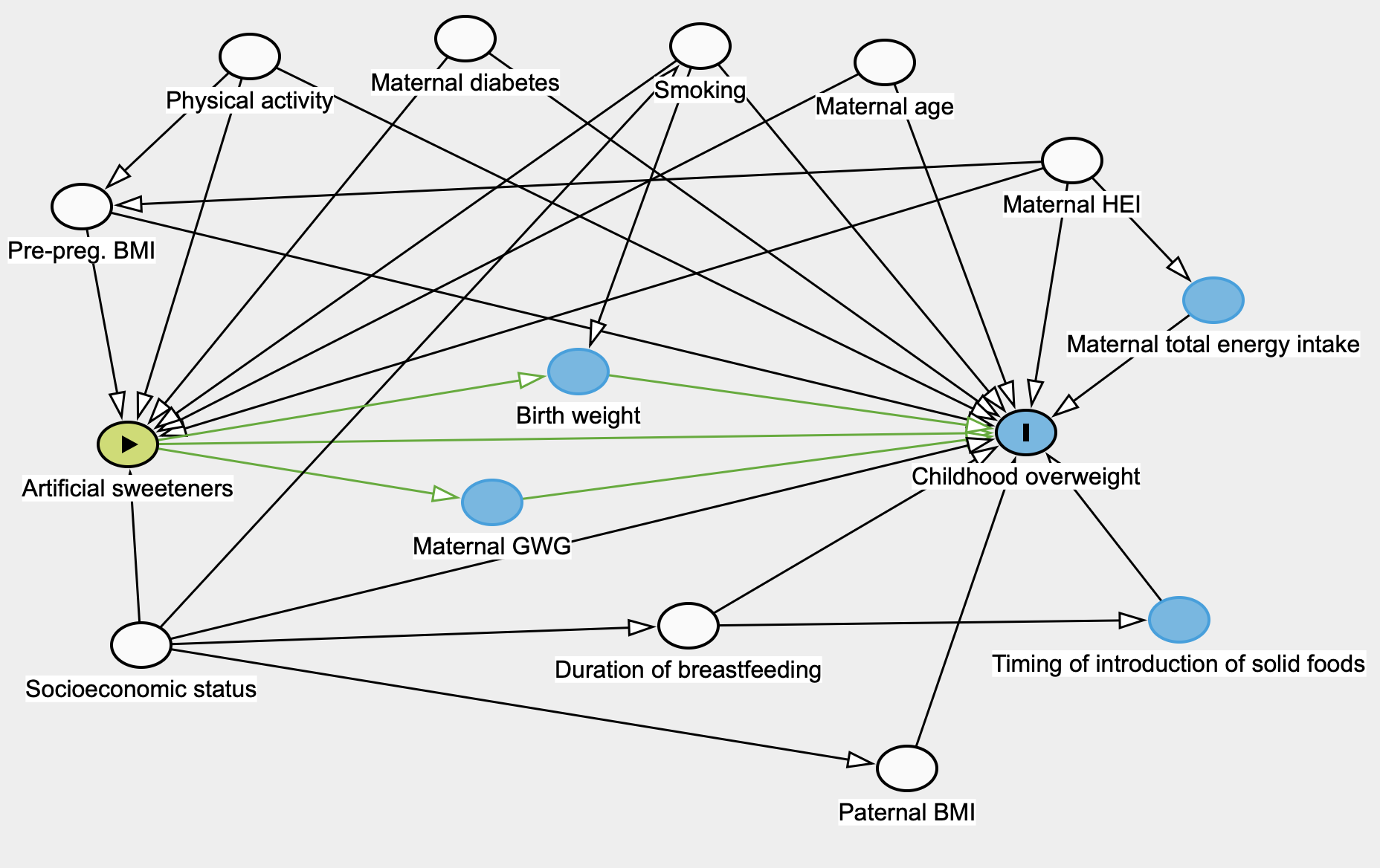

*Figure from dagitty.net*. *Green circle is the exposure, artificial sweeteners. Blue circle with a line is the outcome, childhood overweight. Blue circles are unadjusted variables (gestational weight gain, birth weight, timing of introduction of solid foods and maternal total energy intake) in the primary analysis. White circles are adjusted variables in the primary analysis (Maternal pre-pregnancy body mass index (BMI), age, smoking and physical activity during pregnancy, healthy eating index (HEI), combined socioeconomic status for both parents, paternal BMI, and duration of breastfeeding. Maternal diabetes is counted as adjusted since all pregnancies with a diagnosis of diabetes are excluded from all analyses.*
